# RNF180 Inhibits Proliferation and Promotes Apoptosis of Colorectal Cancer Through Ubiquitination of WISP1

**DOI:** 10.3389/fcell.2020.623455

**Published:** 2021-01-22

**Authors:** Feng Wei, Sang Ba, Mei Jin, Ren Ci, Xuelian Wang, Fusheng E, Ziwen Long

**Affiliations:** ^1^Department of Surgery, Shigatse People's Hospital, Shigatse, China; ^2^Department of Gastric Cancer Surgery, Fudan University Shanghai Cancer Center, Shanghai, China; ^3^Department of Oncology, Shanghai Medical College, Fudan University, Shanghai, China

**Keywords:** colorectal cancer, RNF180, WISP1, 5-fluorouracil, ubiquitination

## Abstract

Colorectal cancer (CRC) is the third leading cause of cancer-related deaths globally and is biologically and clinically heterogeneous. Due to lack of gene expression signatures for risk and prognosis stratification of CRC, identifying novel molecular biomarkers and therapeutic targets may potentially improve CRC prognosis and treatment. RNF180 has been shown to play key contributions to the development of several types of cancers. In the current study, we investigate its role in CRC. In this study, we show that RNF180 expression was significantly downregulated in human CRC tumors and cell lines. Overexpression of RNF180 in CRC cells markedly inhibited cell viability and induced cell apoptosis, while depletion of RNF180 dramatically enhanced cell survival. Moreover, WISP1 was found to be the critical downstream molecule that mediated the tumor suppressive effects of RNF180. Mechanistically, RNF180 ubiquitinated WISP1, resulting in WISP1 downregulation and ultimately leading to suppression of CRC tumor growth in patient-derived xenograft (PDX) mouse models. Last, 5-FU and RNF180 had synergetic effect on the apoptosis induction and tumor growth inhibition. Our findings revealed a crucial role of RNF180 in suppressing tumor growth by ubiquitinating WISP1 in CRC.

## Introduction

Colorectal cancer (CRC) is the third leading cause of cancer deaths in the world, and is the second and fifth leading cause of cancer-related mortalities in United States and China, respectively (Parkin et al., 2001; Chen et al., 2016; Siegel and Miller, 2019). According to the American Cancer Society, the estimated number of CRC cases in the United States for 2019 is 145,600 (Siegel and Miller, 2019). Numerous factors contributing to CRC pathogenesis have been reported, including diet, lifestyle, somatic mutations, and environmental risk factors (Huxley et al., 2009; Markowitz and Bertagnolli, 2009; Aran et al., 2016; Song and Chan, 2019). Currently, the primary treatment options for CRC patients include surgical resection, radiotherapy and chemotherapy. However, the 5-year survival rate for CRC patients with advanced CRC is only 55–75% after surgical resection. Hence, recurrence remains a huge challenge in CRC treatment. To minimize relapse after surgical therapy, 5-Fluorouracil (5-FU)-based chemotherapy has been commonly utilized for treatment. 5-FU administration has been shown to reduce tumor size by ~50% in patients with advanced CRC and to prolong median survival by 5 months. Nonetheless, nearly half of CRC patients with metastasis are resistant to 5-FU-based chemotherapies. Hence, understanding the mechanism for 5-FU resistance and identifying predictive biomarkers are crucial.

WNT1 inducible signaling pathway protein 1 (WISP1, also known as CCN4) is a member of the CCN family of growth factors which regulate diverse cellular functions, including cell proliferation, adhesion, invasion, migration, inflammation, and wound healing (Perbal and Takigawa, 2005; Chen and Lau, 2009; Jun and Lau, 2011). WISP1 was originally discovered as a molecule acting downstream of WNT1 and β-catenin to mediate β-catenin-induced tumorigenesis (Pennica et al., 1998). Previous studies revealed that WISP1 was highly expressed in a variety of cancers, including prostate cancer (Ono et al., 2013; Gaudreau et al., 2019), breast cancer (Chiang et al., 2015), lung cancer (Chen et al., 2015), and melanoma (Deng et al., 2019), suggesting that WISP1 may promote oncogenesis. Although discrepancies exist in CRC studies, as for instance Khor et al. (2006) demonstrated WISP1 enrichment in well-differentiated colorectal tumors while Davies et al. (2010) showed association of higher WISP1 expression with poorly differentiated tumors, there is strong evidence from both studies that WISP1 plays a role in promoting CRC progression and aggressiveness. We and other groups similarly demonstrated an oncogenic role of WISP1 in CRC (Pennica et al., 1998; Stanczak et al., 2011; Wu et al., 2016). However, how WISP1 expression and function in CRC are regulated remains poorly understood.

Given the central role of E3 ubiquitin ligase in diverse cellular processes and diseases, several lines of evidence have linked the function of E3 ligases, especially the RING-type E3 ligases, with colorectal carcinogenesis. Previous studies revealed that RING-type E3 ligases regulate CRC growth through modulation of β-catenin and p53 pathways. Moreover, downregulation of RING finger E3 ligases affected maintenance of genomic stability and cellular homeostasis, permitting tumor formation and development. RING finger protein 180 (RNF180), a RING finger motif containing protein identified in 2008 (Ogawa et al., 2008), has been shown to display tumor suppressive roles in gastric cancer. RNF180 expression was found downregulated in gastric cancer and its re-expression suppressed cell growth and induced apoptosis (Ogawa et al., 2008; Cheung et al., 2012; Deng et al., 2014). However, the function and underlying mechanism of RNF180 in CRC have yet to be explored.

In this study, we set out to determine the therapeutic potential of RNF180 by characterizing its expression, underlying mechanism, and functional significance in CRC pathogenesis. Our findings revealed that RNF180 inhibited CRC tumorigenesis by promoting ubiquitination of WISP. Furthermore, 5-FU and RNF180 had synergetic effect on the apoptosis induction and tumor growth inhibition of CRC, suggesting a novel therapeutic strategy for CRC treatment.

## Materials and Methods

### Bioinformatics Analysis

RNA-seq data related to RNF180 expression in various cancer patients were acquired from The Cancer Genome Atlas (TCGA) dataset, which included 262 cases of colorectal tumor tissues and 41 cases of normal colorectal tissues.

### Clinical Samples

A total of 110 CRC patients in Shigatse People's Hospital were enrolled between October 2012 and March 2014. Detailed information on age, gender, tumor size, disease stage, and histologic differentiation was presented in Table 1. Thirty-five cases of tumor tissues and corresponding non-cancerous tissues were collected and stored at −80°C prior to further analysis. The study was approved by the medical ethics committee of Shigatse People's Hospital and was conducted in accordance with the Declaration of Helsinki. Written informed consents were obtained from all participants.

**Table 1:** Clinicopathological characteristics and follow-up data of 110 patients with colorectal cancer.

**Characteristics**	**Number of patients/number analyzed (%)**
**Age (median, range)**	
≥60	77/110 (70.0%)
<60	33/110 (30.0%)
**Gender**	
Female	43/110 (39.1%)
Male	67/110 (60.9%)
**Tumor size (cm)**	
≥5	63/110 (57.3%)
<5	47/110 (42.7%)
**Stage T**	
T1/T2/T3	96/110 (87.3%)
T4	14/110 (12.7%)
**Stage N**	
N0	64/110 (58.2%)
N1/N2	46/110 (41.8%)
**Stage M**	
M0	73/110 (66.4%)
M1	37/110 (33.6%)
**Stage TNM**	
I/II	62/110 (56.4%)
III/IV	48/110 (43.6%)
**Histologic differentiation**	
Well	65/110 (59.1%)
Poorly	45/110 (40.9%)

### Immunohistochemistry (IHC)

Formalin-fixed paraffin-embedded CRC specimens were used for IHC staining as previously described (Wu et al., 2016). Briefly, tissues were deparaffinized and rehydrated, followed by heat-induced antigen retrieval methods (pH 8.0 EDTA and 3% hydrogen peroxide). The slides were then stained with primary antibodies against RNF180 (ab127548; Abcam, Cambridge, MA, USA; 1:100 dilution) and WISP1 (NBP1-31230; Novus Biologicals, Centennial, CO, USA; 1:100 dilution), followed by horseradish peroxidase (HRP)-conjugated anti-IgG secondary antibodies (D-3004; Long Island Biotech, Shanghai, China; 1:100 dilution). The proportion of tumor cells with positive staining was determined. All patients that had more than 25% of positive cells were classified as high expression group; in contrast, those that had positive cells below 25% were described as low expression group.

### Immunofluorescence (IF) Microscopy

CRC cell lines were fixed with 4% formaldehyde and permeabilized with 0.5% Triton X-100 in PBS. After blocking with 1% bovine serum albumin (BSA) in PBS for 30 min, cells were incubated with anti-RNF180 antibody (H00285671-M05; Novus Biologicals, 1:1000 dilution) and anti-WISP1 antibody (NBP1-31230; Novus Biologicals; 1:1000 dilution), and further treated with either Alexa Fluor 555-labeled Donkey Anti-Rabbit IgG (H+L) antibody (A0453; Beyotime Biotechnology, Shanghai, China; 1:500 dilution) or Alexa Fluor 488-labeled Goat Anti-Mouse IgG (H+L) antibody (A0428; Beyotime Biotechnology; 1:500 dilution), respectively. Cells were stained for nuclei with DAPI-containing hard-set media (C1002; Beyotime Biotechnology; 1:500 dilution). Fluorescence images were taken by laser-scanning confocal microscopy (Leica Microsystems, Wetzlar, Germany).

### Cell Cultures

Human CRC cell lines (SW620, LOVO, RKO), and fetal colon cell line (FHC) were obtained from the cell bank of Shanghai Biology Institute, Chinese Academy of Science (Shanghai, China) and maintained at 37°C in a 5% CO_2_ incubator. SW620 and LOVO cells were maintained in RPMI-1640 media (Life Technologies, Carlsbad, CA, USA), supplemented with 10% fetal bovine serum (FBS) and 1% Pen/Strep (Life Technologies). RKO cells were cultured in MEM media (Life Technologies) containing 10% FBS and 1% Pen/Strep. FHC cells were grown in DMEM/F-12 media (Life Technologies) containing 10 mM HEPES, 10 ng/ml cholera toxin, 0.005 mg/ml insulin, 0.005 mg/ml transferrin, 100 ng/ml hydrocortisone, 20 ng/mL human recombinant EGF, 10% FBS, and 1% Pen/Strep.

### Gene Overexpression and Knockdown

RNF180 and WISP1 overexpression plasmids were constructed by cloning the coding sequences of RNF180 or WISP1 into pLVX-Puro vectors (Takara Bio Inc., Mountain View, CA, USA). To generate knockdown clones, synthesized shRNA oligos targeting RNF180 (shRNA#1, 5′-GGAGTATCTTGAGAATCAA-3′; shRNA#2, 5′-GCATTAATCAGAGGCTTAA-3′; shRNA#3, 5′-GGATGGATTACCTGCACTT-3′) or WISP1 (shRNA#1: GGACATCCATACACTCATT; shRNA#2: CCCAAGTACTGTGGAGTTT; shRNA#3, CCCTGACTTCTCAGAAATT) were cloned into pLKO.1 plasmids (Addgene, Cambridge, MA, USA). Recombinant plasmids, together with packaging/envelope plasmids psPAX2 and pMD2.G, were co-transfected into 293T cells using Lipofectamine 2000 (Invitrogen, Carlsbad, CA, USA) following manufacturer's instructions. 48 h after transfection, virus particles were collected and transfected into cells of interest to generate overexpression and knockdown cell lines. Cells transfected with scramble shRNA (shNC) or blank plasmid (vector) were used as negative controls.

### Cell Proliferation Assay

Cell proliferation was analyzed using Cell Counting Kit-8 (CCK-8) (SAB, College Park, MD, USA) according to the manufacturer's instructions. Briefly, SW620, LOVO, and RKO cells transduced with the indicated plasmids were plated in 96-well plates (3,000 cells/well) and incubated at 37°C overnight, followed by CCK-8 incubation at 37°C for 1 h. The optical density (OD) at 450 nm was determined using a multi-mode plate reader (DNM-9602, Perlong Medical Co., Beijing, China).

### Cell Apoptosis Assay

SW620, LOVO, and RKO cells were grown in six-well plates (5 x 10^5^ cells/well) until they reached 50% confluence. Then, cells transduced with the indicated plasmids were treated with or without 5-FU (2 μM) or vehicle control for 24 h. Following incubation with 5 μl of fluorescein isothiocyanate-labeled annexin V (Annexin V-FITC) and 5 μl of propidium iodide (PI), cell apoptosis was assessed by flow cytometry (FACSArial I, BD Biosciences, San Jose, CA, USA). Cells that were FITC Annexin V positive and PI negative were considered apoptotic.

### Quantitative Real Time PCR (Q-PCR)

Total RNA was extracted from CRC tissues and cell lines using TRIzol reagent (Life Technologies) and reverse transcribed into cDNA with PrimeScript kit (Takara Biotechnology, Dalian, China) according to the manufacturers' instructions. Quantitative PCR was conducted using SYBR green PCR master mix (Applied Biosystems, Foster, CA, USA) on ABI 9700 real-time PCR system (Applied Biosystems). The following primers were used: RNF180-F: 5′-AGT TAC AAG AAG GCA GTT CC-3′, RNF180-R: 5′-AAT CCA ATG ACC CAG TTC AC-3′; WISP1-F: 5′-GGA TTG TCT GGC AGT AGC C-3′, WISP1-R: 5′-GAA GCA GTC AGC CCT TAT G-3′; GAPDH-F: 5′-AAT CCC ATC ACC ATC TTC-3′, GAPDH-R: 5′-AGG CTG TTG TCA TAC TTC-3′. The fold changes of mRNA were calculated and normalized to GAPDH with 2^−ΔΔ*CT*^ method.

### Western Blot

RIPA lysis buffer containing protease inhibitor cocktail (Sigma, St. Louis, MO, USA) was used for protein extraction. 30 μg of proteins were separated on SDS-PAGE gels, followed by transfer onto nitrocellulose membranes (MilliporeSigma, Burlington, MA, USA). Membranes were blocked with 5% skim milk and incubated at 4°C overnight with primary antibodies anti-RNF180 (ab127548, Abcam, Cambridge, MA, USA); anti-WISP1 (ab155654, Abcam); anti-Ki67 (ab92742, Abcam); anti-cleaved Caspase-3 (ab2302, Abcam); anti-cleaved Caspase-9 (ab2324, Abcam); anti-GAPDH (Cell Signaling Technology, Danvers, MA, USA) followed by incubation with HRP-conjugated secondary antibody (Beyotime, Shanghai, China). Membranes were visualized using ChemiDoc Imaging Systems (Bio-Rad, Richmond, CA, USA).

### Tandem Affinity Purification

293T cells stably expressing FLAG-RNF180 were generated. Cells from 50 10-cm^2^ culture dishes were collected and lysed with pre-cooled RIPA lysis buffer (20 mM Tris pH7.5, 150 mM NaCl, 1% Triton X-100). The lysate was collected, diluted with the same volume of ddH_2_O and incubated with anti-FLAG magnetic beads (Sigma-Aldrich) overnight at 4°C. The bound proteins were eluted twice with FLAG peptide by incubating for 1 h at 4°C, resolved on SDS-PAGE, and stained with Coomassie Brilliant Blue. The entire lane primarily in RNF180 overexpressed cells was excised and LC/MS identification using a mass spectrometer (Thermo Scientific Q Exactive) as previously described (Xie et al., 2020).

### Protein Stability

To evaluate protein stability, RKO cells transduced with the indicated plasmids were treated with 100 μg*/*mL cycloheximide (CHX; Merck Millipore, Germany) during indicated times and harvested. Protein quantity of WISP1 was then determined by western blot analysis.

### Co-immunoprecipitation (Co-IP) and Ubiquitination Assay

Cell lysates extracted with RIPA buffer were incubated with anti-RNF180 (NB100-56179; Novus Biologicals), anti-WISP1 (sc-133198; Santa Cruz Biotechnology, Santa Cruz, CA, USA), or normal IgG antibody (sc-2027; Santa Cruz Biotechnology) at 4°C overnight, followed by incubation with Protein A/G PLUS-Agarose beads (sc-2003; Santa Cruz Biotechnology, Inc.) at 4°C for 2 h. The immunocomplexes were washed three times with lysis buffer on a magnetic rack, and then examined by immunoblotting with anti-RNF180 (ab127548; Abcam), anti-WISP1 (ab155654; Abcam), and anti-ubiquitin (ab7780; Abcam) antibodies.

### His-Ubiquitin Pull-Down Assay

Full-length WISP1 was cloned into pCMV-Tag 2B vector, and mutations were introduced into WISP1 with QuikChange II Site-directed Mutagenesis kit (Agilent Technologies, Santa Clara, CA, USA). The generated plasmids were designated as Flag-WISP1 (WT), Flag-WISP1 (K50R), Flag-WISP1 (K190R), and Flag-WISP1 (K268R). RNF180 sequence containing a myc tag was obtained from GENEWIZ, lnc. (Suzhou, China) and further cloned into p-DONR221 vector for expression. Human ubiquitin with 6xHis tag was cloned into pcDNA-DEST40. All DNA constructs were sequence verified. Wild-type WISP1 (WT) or WISP1 mutants were transfected into HEK293T cells, along with myc-RNF180 and His-Ub constructs using Lipofectamine 2000 reagent according to manufacturer's instructions. 48 h later, cells were lysed and incubated with Ni-NTA agarose beads (Qiagen, Hilden, Germany). The complexes were washed, eluted, and applied onto SDS-PAGE gels for immunoblotting.

### *In vivo* Studies

RKO cells (5 × 10^6^ cells) overexpressing RNF180, WISP1 or both were subcutaneously injected into the armpits of 4–5-week-old male nude mice (Shanghai Laboratory Animal Company, Shanghai, China) (*n* = 6 per group). Tumors were measured every 3 days with a digital caliper and tumor volumes were determined using the formula: Tumor volume = (length × width^2^)/2. Thirty-three days after injection, mice were euthanized, tumor characteristics were recorded and xenografts were collected for further analysis.

To establish patient-derived xenograft (PDX) model, tumor tissues (F0) from CRC patients were collected at the time of surgery at Shanghai Jiao Tong University Affiliated Sixth People's Hospital, divided into two groups. One group was fixed for detection of RNF180 expression in IHC staining, and the remaining fresh tumor tissues were cut into 2-mm^3^ pieces and subcutaneously transplanted into 6–8-week-old nude mice within 1 h of removal of tissues. By palpation of the skin at the tumor site, we selected mice that bore tumor nodules and began to measure the tumor volumes. When the tumor size reached 100–200 mm^3^, the samples (F1) were divided into pieces for *in vivo* passaging to construct F2 and then F3 tumors as described above. When the F3 tumor size reached 100 mm^3^, the mice bearing tumors were randomly divided into DMSO control and 5-FU consisting of five mice per group. 5-FU (50 mg/kg; once per week) chemotherapy was initiated at day 12. Thirty-three days after transplantation, mice were sacrificed, and tumor size was measured. Laboratory animal care and experimentation were performed in conformity with the animal ethics guidelines, with protocols approved by the Shigatse People's Hospital.

### Statistical Analysis

Three biological replicates and three technical replicates were used in the analysis. Data are expressed as the mean ± SD of three independent experiments and analyzed by GraphPad Prism 7.0 (GraphPad Software, San Diego, CA, USA). Comparison between different experimental groups was achieved with ANOVA or Student's *t*-test. *P* < 0.05 was considered to be statistically significant, and the significance was interpreted as follow unless specified otherwise: *P < 0.05; **P < 0.01; ***P < 0.001. Kaplan-Meier estimator and Cox's proportional hazards regression model were used to calculate overall survival (OS), disease-free survival (DFS), and differences were analyzed by log-rank test.

## Results

### RNF180 Was Downregulated in Human Colorectal Cancer (CRC)

**Figure 1:**
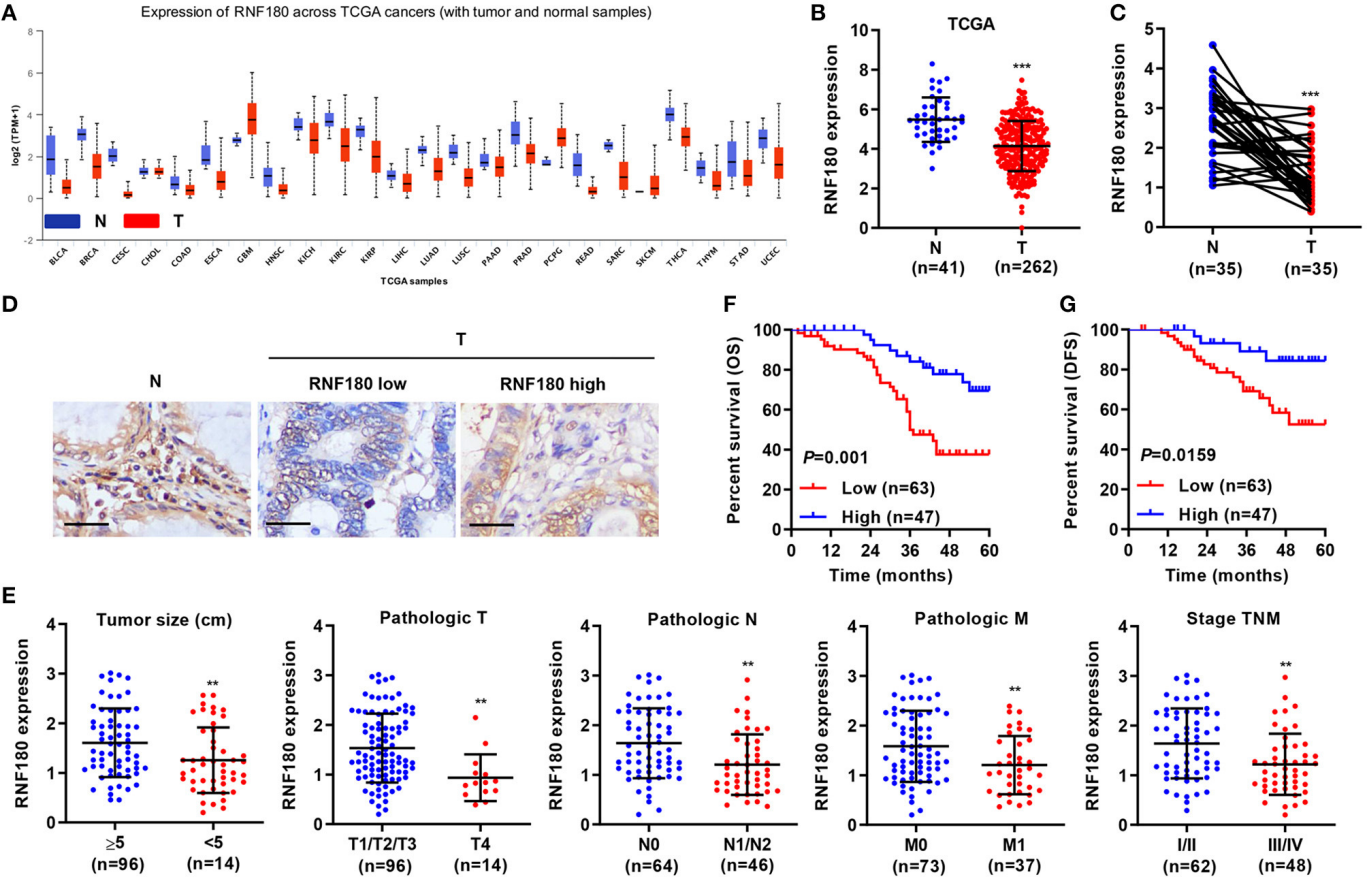
Relationship between clinical factors and RNF180 expression in CRC. **(A)** The mRNA expression of RNF180 was evaluated in patient samples from 24 TCGA projects. **(B)** RNF180 expression in 262 CRC tissues and 41 normal tissues acquired from TCGA RNA-seq datasets. **(C)** RNF180 expression was assessed via quantitative RT-PCR in 35 pairs of CRC samples collected at our hospital. **(D)** Representative images of immunohistochemistry (IHC) staining in CRC samples collected at our hospital, showing differential expression of RNF180. Scale bar: 50 μm. **(E)** Relationship between clinical factors and RNF180 expression in CRC. **(F,G)** Kaplan-Meier curves of overall survival (OS) **(F)** and disease-free survival (DFS) **(G)** of CRC patients based on RNF180 expression. **P < 0.01, ***P < 0.001.

To explore the role of RNF180 in tumorigenesis, we utilized RNA-seq data from the Cancer Genome Atlas (TCGA) and evaluated RNF180 expression. As shown in Figure 1A, the expression of RNF180 was remarkably lower in various cancers (*p* < 0.05 in all projects except SKCM). Focusing on colorectal tumor samples, we confirmed that RNF180 was downregulated in TCGA colorectal tumor samples compared to normal colorectal tissues (Figure 1B). The expression of RNF180 was also significantly reduced in 35 pairs of CRC patient samples from a hospital cohort (Figure 1C). Immunohistochemistry (IHC) staining of RNF180 in CRC tissues was carried out, and tumor samples were characterized as RNF180 low or RNF180 high groups for further analyses (Figure 1D). Furthermore, clinicopathological features of patients were analyzed based on the expression of RNF180. Remarkably, lower expression of RNF180 in patients was accompanied with larger tumor size (*p* = 0.0087), advanced overall pathological stage (T, N, and M) (*p* = 0.0025, 0.0011, 0.0065, respectively), and advanced TNM stage (*p* = 0.0014) (Figure 1E), whereas the expression of RNF180 was not associated with patient age, gender, or histologic differentiation (data not shown). Furthermore, lower expression of RNF180 protein was significantly associated with poor patient overall survival (OS) and disease-free survival (DFS) (Figures 1F,G). Taken together, these results suggest that RNF180 may function as a tumor suppressor in the pathogenesis of CRC.

### RNF180 Inhibited Cell Proliferation and Promoted Apoptosis in CRC Cells

**Figure 2:**
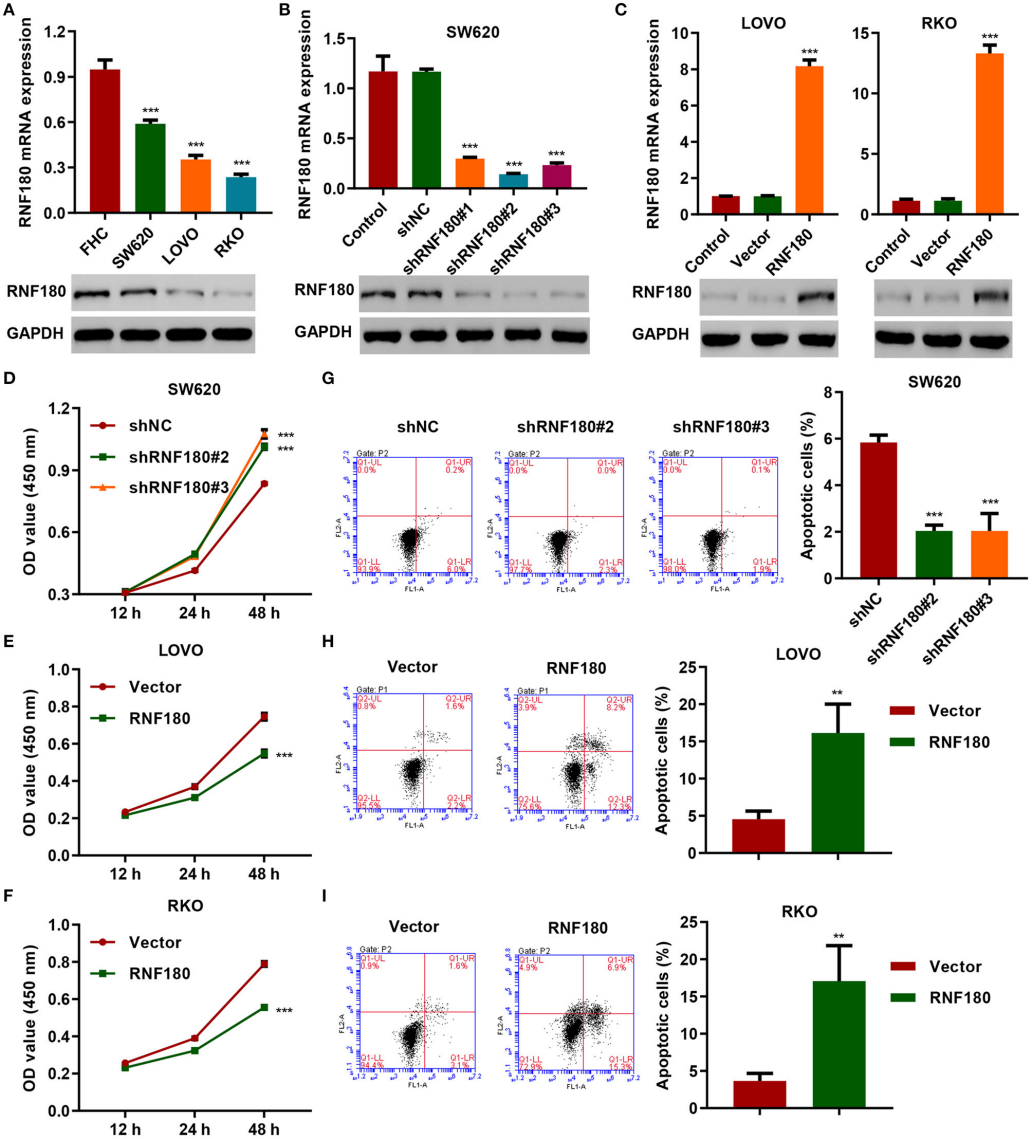
RNF180 silencing promoted cell proliferation while enforced RNF180 overexpression exhibited the opposite effect. **(A)** mRNA expression and protein levels of RNF180 in CRC cell lines and fetal colon cell line FHC were determined by quantitative RT-PCR and Western blot, respectively. **(B)** RNF180 was silenced using shRNA hairpins in SW620 cells, and knockdown efficiency was validated. **(C)** RNF180 was overexpressed in both LOVO and RKO cells, and RNF180 overexpression was confirmed. **(D–I)** Cell proliferation assay **(D–F)** and apoptosis assay **(G–I)** were measured in cells that were transduced with the indicated plasmids, and apoptotic cells were quantified as bar graphs. **P < 0.01, ***P < 0.001 compared with FHC, shNC or vector.

To determine the cellular functions of RNF180, we employed three CRC cell lines: SW620, LOVO, and RKO. Consistently, RNF180 was downregulated in all three types of cells, at both mRNA and protein levels, compared to a fetal colon cell line FHC. SW620 cells exhibited relatively higher expression of RNF180 while LOVO and RKO cells displayed subtle expression (Figure 2A). Hence, we carried out RNF180 knockdown by shRNA in SW620 cells (Figure 2B), and RNF180 overexpression in LOVO and RKO cells (Figure 2C). Expression of RNF180 was confirmed accordingly. Interestingly, RNF180 silencing in SW620 cells significantly promoted cell proliferation, as measured by CCK-8 assay (Figure 2D). In contrast, RNF180 overexpression inhibited cell proliferation in LOVO and RKO cells (Figures 2E,F). Moreover, RNF180 knockdown in SW620 cells reduced apoptosis as shown by flow cytometry (Figure 2G), whereas RNF180 overexpression significantly elevated apoptosis in LOVO and RKO cells (Figures 2H,I). Collectively, these results support that RNF180 played tumor suppressive functions.

### RNF180 Interacted With WISP1 and Ubiquitinated WISP1 at K50

**Figure 3:**
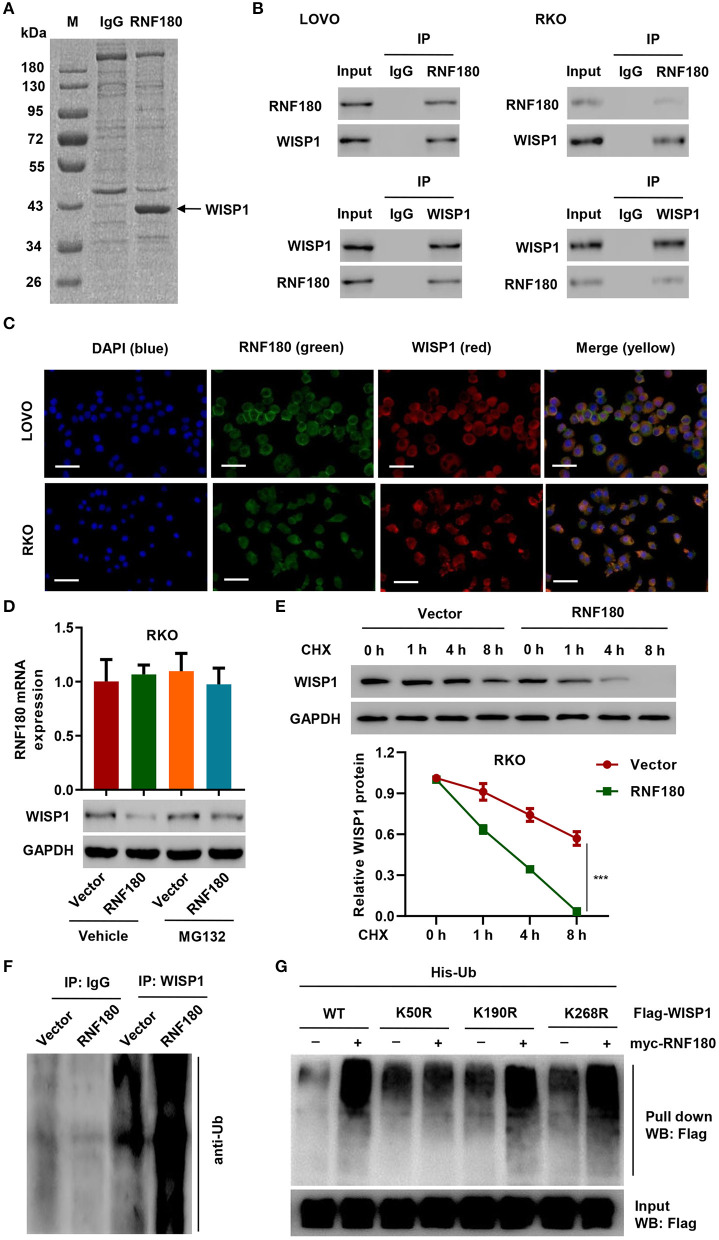
RNF180 interacted with WISP1 and induced K50-linked ubiquitination of WISP1. **(A)** Purification of RNF180 immunocomplex. Proteins were separated on SDS-PAGE and stained by Coomassie Blue. **(B)** Immunoprecipitation was carried out with IgG control, anti-RNF180, or anti-WISP1 antibody. The immunoprecipitants were then incubated with the indicated antibodies. **(C)** Subcellular localization of WISP1 (red) and RNF180 (green) was measured by immunofluorescence. DAPI (blue) was used for nuclear counterstain. Scale bar: 50 μm. **(D)** RKO cells overexpressing RNF180 were treated with MG132 (10 μM), and the mRNA and protein levels of WISP1 were assessed by quantitative RT-PCR and Western blot. **(E)** RKO cells transduced with the indicated plasmids were treated with CHX (100 μg/mL), and WISP1 expression was determined by western blot analysis. **(F)** RNF180-overexpressing RKO cells were immunoprecipitated with WISP1 or IgG antibodies and ubiquitination was evaluated by Western blot. **(G)** HEK293T cells were co-transfected with either the wild-type Flag-WISP1 (WT) or mutants (K50R, K190R, and K268R), together with myc-RNF180 and His-Ub plasmids, and pull down assay was performed. ***P < 0.001.

To investigate how RNF180 functions in CRC, we identified candidate proteins associated with RNF180 by Co-IP assay and proteomics analysis. There is one specific band in the anti-RNF180 group compared to anti-IgG group (Figure 3A). We further excised and analyzed the band by LC/MS and identified potential binding proteins. Among the proteins identified may be associated with RNF180 protein, WISP1, which was highly expressed in color cancer and associated with apoptosis inhibition, cell invasion, and inferior prognosis through directly bound to β-catenin (Wu et al., 2016), was selected for further investigation. Hence, we further explored the interaction between RNF180 and WISP1 in CRC cells. In both LOVO and RKO cells, co-immunoprecipitation using anti-RNF180 antibody confirmed direct interaction between RNF180 and WISP1, and similar result was observed with anti-WISP1 antibody (Figure 3B). To examine the subcellular distribution of the two proteins, immunofluorescence microscopy was performed. As shown in Figure 3C, the overall distribution of RNF180 and WISP1 was similar, and both RNF180 and WISP1 co-localized in the cytoplasm of cells.

To evaluate RNF180-mediated ubiquitination of WISP1, we overexpressed RNF180 in RKO cells. Western blot revealed that overexpression of RNF180 reduced the protein level of WISP1, whereas this effect was abrogated when cells were treated with 10 μM of proteasome inhibitor MG132 for 4 h, suggesting that RNF180 induced degradation of ubiquitin-conjugated WISP1 (Figure 3D). To further establish that RNF180 regulates WISP1 stability, we treated RKO cells with CHX and determined the half-life of WISP1. As shown in Figure 3E, WISP1 stability was dramatically decreased in RKO cells with RNF180 overexpression. These results demonstrate that RNF180 stabilizes WISP1. In addition, Western blot indicated ubiquitination enrichment in RNF180-overexpressing RKO cells following immunoprecipitation with WISP1 or IgG antibodies (Figure 3F).

By using bio-computer analysis (http://www.ubpred.org/), three Lys residues at positions 50, 190, and 268 of WISP1 were predicted as potential ubiquitination sites. These Lys residues were then mutated to Arg. HEK293T cells were co-transfected with either wild-type Flag-WISP1 (WT) or mutant WISP1 (K50R, K190R, and K268R), together with myc-RNF180 and His-Ub plasmids, followed by a pull down assay using His-tag antibody. Strikingly, although ubiquitination of WISP1 in WT and K190R/K268R mutants was elevated upon RNF180 overexpression, ubiquitination in K50R mutant was not affected, suggesting that Lys50 was essential for RNF180 mediated WISP1 ubiquitination (Figure 3G).

### RNF180 Alleviated the Oncogenic Potential of WISP1

**Figure 4:**
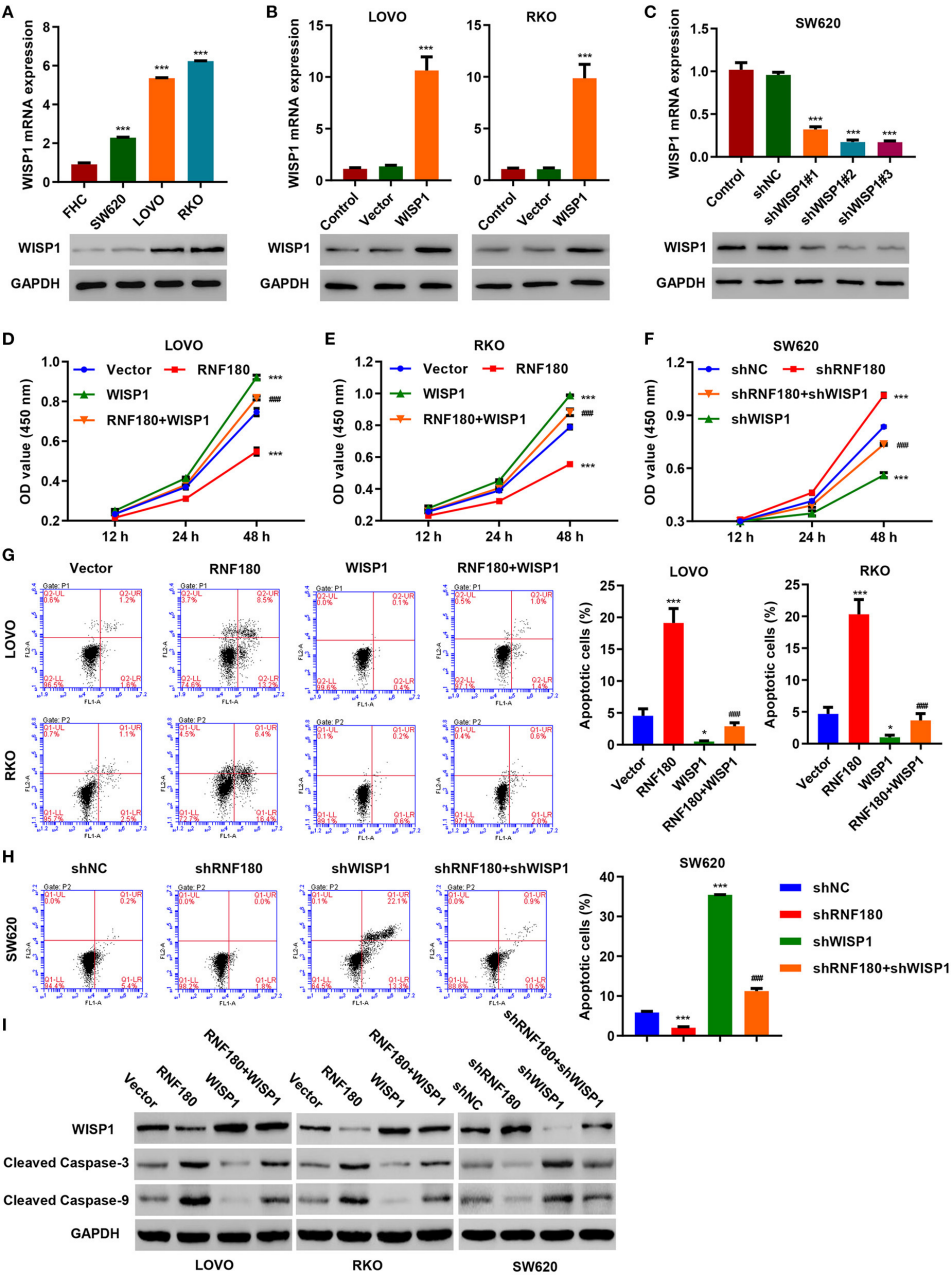
WISP1 neutralized RNF180-mediated effects on cell proliferation and apoptosis. **(A)** mRNA expression and protein levels of WISP1 in CRC cell lines and fetal colon cell line FHC were determined by quantitative RT-PCR and Western blot, respectively. **(B)** WISP1 was overexpressed in both LOVO and RKO cells, and WISP1 overexpression was confirmed. **(C)** WISP1 was silenced using shRNA hairpins in SW620 cells, and knockdown efficiency was validated. CRC cells were transduced with the indicated plasmids, and cell proliferation assay **(D–F)**, apoptosis assay **(G,H)**, and immunoblotting **(I)** were carried out. *P < 0.05, ***P < 0.001 compared with shNC or vector. ^###^*P* < 0.001 compared with RNF180.

To further confirm regulation of WISP1 by RNF180, we first employed three CRC cell lines and overexpressed WISP1 in LOVO and RKO cells, and knocked down WISP1 in SW620 cells. Consistently, WISP1 protein levels were upregulated in all three types of CRC cell lines compared to a fetal colon cell line FHC. LOVO and RKO cells exhibited relatively higher expression of WISP1 while SW620 cells displayed subtle expression (Figure 4A). Expression of WISP1 in LOVO, RKO and SW620 was confirmed accordingly (Figures 4B,C). Consistent with our previous study (Wu et al., 2016), overexpression of WISP1 enhanced CRC cell proliferation (Figures 4D,E), whereas silencing of WISP1 in SW620 cells significantly reduced proliferation (Figure 4F). Remarkably, overexpression of RNF180 attenuated WISP1-induced cell proliferation (Figures 4D,E), and silencing of RNF180 alleviated the reduction in cell proliferation caused by WISP1 knockdown (Figure 4F). Similarly, RNF180 alleviated the effects of WISP1 on cell apoptosis (Figures 4G,H). Consistent with this, expression of both cleaved Caspase-3 and cleaved Caspase-9 was increased upon RNF180 overexpression and WISP1 knockdown, further confirming the neutralization effects caused by RNF180 (Figure 4I). Taken together, our data demonstrate that RNF180 regulates CRC cell proliferation and apoptosis via modulation of WISP1.

### RNF180 Regulated WISP1-Induced Tumor Formation *in vivo*

**Figure 5:**
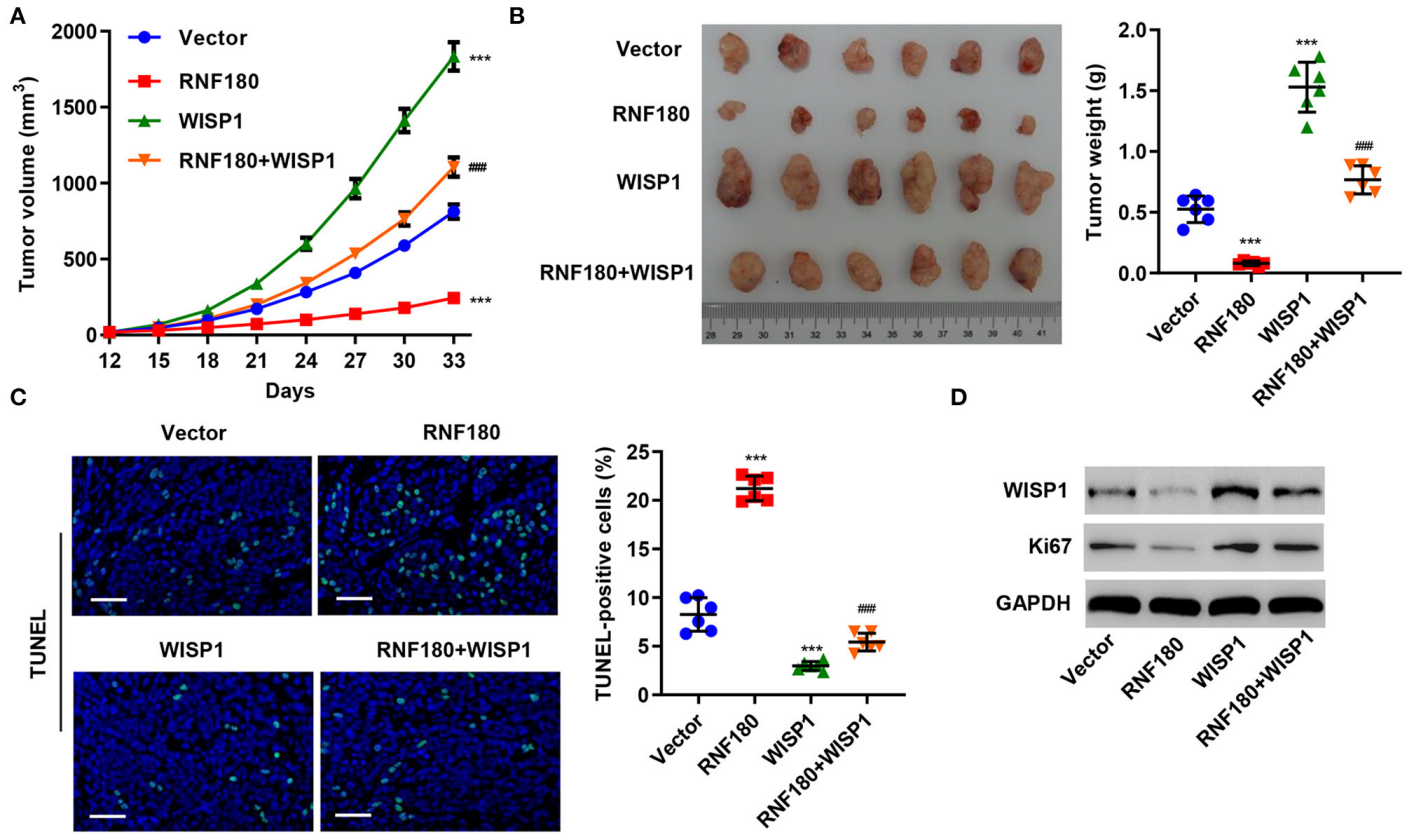
RNF180 antagonized WISP1 in regulating tumor growth in mouse. RKO cells stably expressing the indicated genes were subcutaneously injected into nude mice (*n* = 6 per group). **(A)** Tumor volume was measured every 3 days for 33 days. **(B)** At day 33, mice were euthanized, and tumor characteristics were recorded. **(C)** Representative images of TUNEL staining in xenograft mouse tumors. Apoptotic cells were quantified accordingly. Scale bar: 50 μm. **(D)** Protein levels of WISP1 and Ki67 in RKO cells that were transduced with the indicated plasmids were measured by Western blot. ***P < 0.001 compared with vector. ^###^*P* < 0.001 compared with RNF180.

To further assess the function of RNF180 on WISP1 *in vivo*, we employed RKO cell xenograft mouse model. RKO cells stably expressing RNF180, WISP1, or both were injected subcutaneously into nude mice (*n* = 6 per group) and tumor development was evaluated. As shown in Figure 5A, WISP1 overexpression significantly facilitated tumor formation. In contrast, RNF180 overexpression significantly reduced tumor volume. Notably, when cells were transduced with both WISP1 and RNF180, RNF180 expression reversed the effects of WISP1 on promoting tumor growth. Correspondingly, tumor weights exhibited the same pattern, with RNF180+WISP1 mice exhibiting intermediate tumor size (Figure 5B). Consistent with tumor growth results, TUNEL staining showed dramatically increased apoptotic cells in mice with RNF180 overexpression, whereas no significant apoptosis alteration was observed in RNF180+WISP1 mice compared to control mice (Figure 5C). The expression of WISP1 and Ki67 was confirmed in RKO cells that were transduced with the indicated plasmids (Figure 5D).

### 5-FU and RNF180 Had Synergetic Effect in CRC

**Figure 6:**
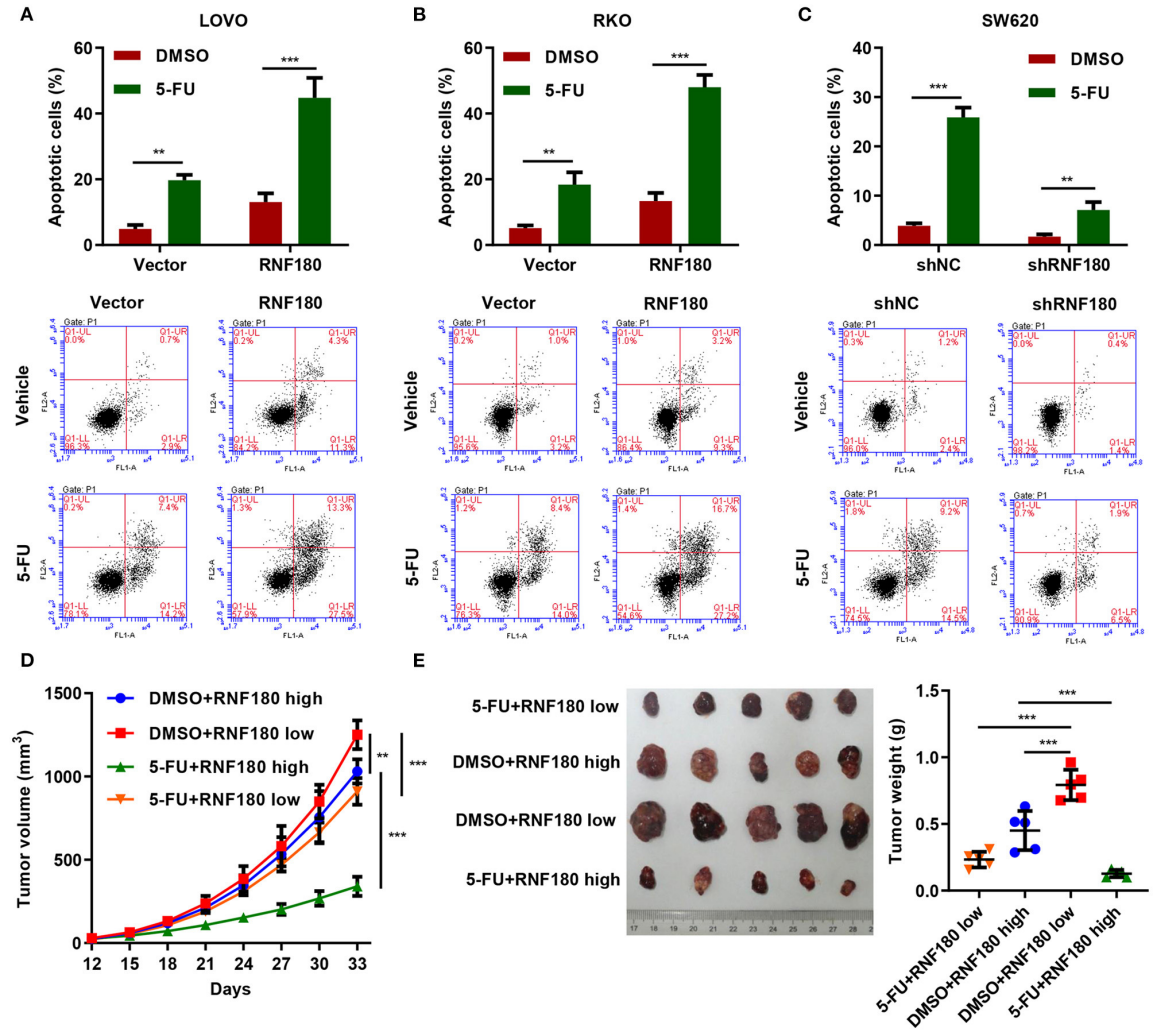
5-FU and RNF180 had synergetic effect in CRC. CRC cells transduced with the indicated plasmids were treated with 2 μM of 5-FU or vehicle for 24 h. **(A–C)** Cell apoptosis was detected by flow cytometry and quantified accordingly. **(D,E)** Tumor volume and weight were measured in mice with patient-derived xenograft (PDX) models following 5-FU chemotherapy (*n* = 5 per group). **P < 0.01, ***P < 0.001.

5-Fluorouracil (5-FU) has been the first-choice chemotherapy drug for CRC for many years (Longley et al., 2003). To understand the therapeutic potential of RNF180, CRC cells were transduced with the indicated plasmids and treated with 2 μM of 5-FU or DMSO for 24 h. Consistently, LOVO and RKO cells with RNF180 overexpression exhibited increased apoptotic induction in DMSO control. Moreover, 5-FU exhibited highest apoptotic rate in LOVO and RKO cells with RNF180 overexpression (Figures 6A,B). In contrast, 5-FU exhibited lowest apoptotic rate in SW620 cells with RNF180 knockdown (Figure 6C).

We further established CRC patient-derived xenograft (PDX) models by subcutaneously transplanting CRC patient tumor fragments to mice. Based on the expression of RNF180 in CRC patients, the PDX mice were split into two groups: RNF180 high vs. RNF180 low. 5-FU (50 mg/kg; once per week) chemotherapy and vehicle treatment were initiated 12 days after transplantation (*n* = 5 per group) and mice were sacrificed at day 33 for analysis. Consistently, lower expression of RNF180 resulted in increased tumor volume in vehicle groups. In RNF180 low groups, 5-FU showed relatively potent antitumor activity. Remarkably, 5-FU exhibited highest antitumor activity in RNF180 high groups (Figure 6D). Correspondingly, tumor weights displayed similar results, with the 5-FU+RNF180 high group showing smallest tumors (Figure 6E).

### RNF180 and WISP1 Were Negatively Correlated in CRC Patient Samples

**Figure 7:**
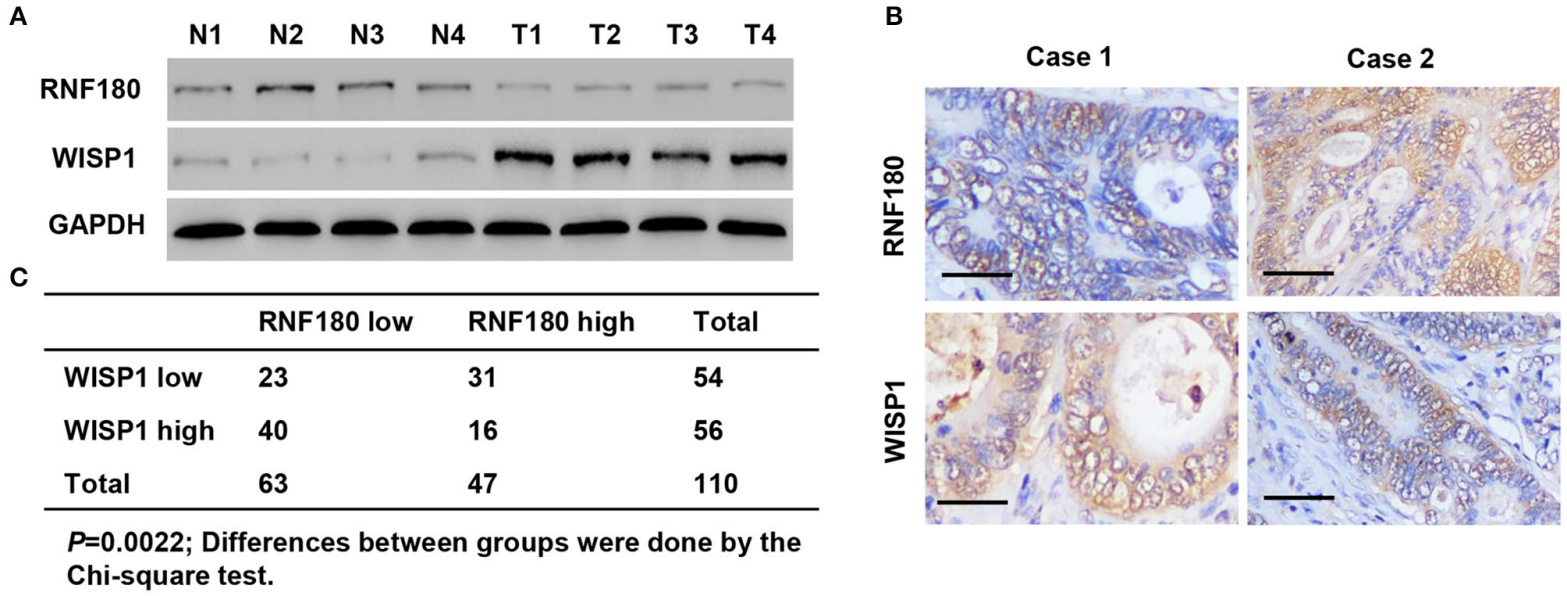
RNF180 expression was correlated with WISP1 in clinical CRC samples. **(A)** Protein levels of RNF180 and WISP1 in CRC patient tumor samples (T1**–**T4) and corresponding non-cancerous tissues (N1**–**N4) via Western blot from hospital cohort. **(B)** Representative images of immunohistochemical staining of RNF180 and WISP1 in CRC samples. Scale bar: 50 μm. **(C)** Correlation analysis of RNF180 and WISP1 in CRC samples.

To explore the relationship between RNF180 and WISP1 in CRC clinical samples, we performed Western blot on tissues collected at our hospital. As shown in Figure 7A, RNF180 expression was lower in CRC tissues (T1–T4), where WISP1 was highly expressed, indicating a negative correlation between RNF180 and WISP1. We also conducted IHC staining on CRC tissues and scored RNF180 and WISP1 as low or high (Figure 7B). Of the 110 cases stained successfully for both RNF180 and WISP1, RNF180 staining was significantly different between WISP1-low and WISP1-high cases, with 31/54 (57.4%) of WISP1-low and 16/56 (28.6%) of WISP1-high cases being RNF180 high, and the difference was significantly different (Figure 7C).

## Discussion

Our studies revealed that Ring Finger Protein 180 (RNF180) was downregulated in a wide range of cancers, including colorectal cancer (CRC). Based on our analyses of the anti-tumor activity of RNF180, we found that RNF180 inhibited the expression of the WISP1 oncoprotein by promoting WISP1 ubiquitination, thereby suppressing tumor growth. In addition, 5-FU and RNF180 had synergetic effect on the apoptosis induction and tumor growth inhibition of CRC cells *in vitro* and *in vivo*. To our knowledge, our studies are the first to demonstrate the anti-oncogenic potential of RNF180 in CRC.

However, the molecular mechanism of CRC remains intricate. Accumulating evidence pointed that E3 ubiquitin ligase, the most important element of the ubiquitin-proteasome system (UPS), was involved in the development of cancers by directly binding to target proteins, including oncogenes and tumor suppressors, and triggering target degradation (Hoeller et al., 2006; Nakayama and Nakayama, 2006). Our study revealed that RNF180 co-localized with WISP1 and subsequently ubiquitinated WISP1, targeting it for degradation, to execute tumor suppressive effects in CRC. However, whether RNF180 could also be involved in other cancers remains to be determined. Intriguingly, RNF180 cannot eliminate the effects of WISP1 completely, implying that there might be alternative mediators or pathways involved in WISP1 signaling. Future studies will need to focus on the identification of these alternative pathways.

Beyond uncovering tumor suppressive functions of RNF180 and its mechanism of regulation on WISP1, our findings have presented a novel therapeutic strategy to restore RNF180/WISP1 pathway for CRC treatment. First, our studies revealed that the expression of RNF180 was encumbered in CRC and was negatively correlated with overall survival in CRC patients, suggesting that RNF180 may function as a prognostic biomarker for aiding the enrollment of appropriate patients and guiding treatment decisions. Second, RNF180 induction led to WISP1 ubiquitination, resulting in suppression of tumor growth both *in vitro* and *in vivo*. Our observations are consistent with previous findings in gastric cancer, in which RNF180 acts as a novel potential tumor suppressor in gastric carcinogenesis (Ogawa et al., 2008; Cheung et al., 2012; Deng et al., 2014). Thus, RNF180 may have a broader role and potential as a therapeutic target for cancer treatment.

Additional supporting evidence for the importance of RNF180 can be gleaned from the synergetic effect of RNF180 and 5-FU. Although 5-Fluorouracil (5-FU) chemotherapy has often been used for treatment of CRC (Grem, 2000; Longley et al., 2003; Andre et al., 2004), the prognosis is very poor as about 50% of colorectal patients are resistant to 5-FU based chemotherapy (Mader et al., 1998; Zhang et al., 2008). Using the CRC PDX mouse, we demonstrated that 5-FU significantly reduced tumor burden in the group with higher RNF180 expression. Intriguingly, previous studies have linked WISP1 expression to establishing resistance to radiotherapy and chemotherapy (Zhang et al., 2015; Klee et al., 2016). However, additional studies are needed to further validate the role of WISP1 in chemosensitivity in CRC.

Collectively, using *in vitro* assessment of mechanism and follow-up studies using animal models, our findings not only revealed a promising role for RNF180 as a prognostic biomarker for guiding treatment decisions, but also proposed a potential strategy for targeting WISP1. Thus, RNF180/WISP1 pathway intervention represents an attractive avenue for exploring future cancer therapies.

## Data Availability Statement

The original contributions generated for the study are included in the article/supplementary material, further inquiries can be directed to the corresponding author.

## Ethics Statement

The studies involving human participants were reviewed and approved by Shigatse People's Hospital. The patients/participants provided their written informed consent to participate in this study. The animal study was reviewed and approved by Shigatse People's Hospital.

## Author Contributions

FW and SB conceived and designed the work. SB, MJ, and RC performed the research, and collected and analyzed the data. XW and FE collected human tissue samples. RC, XW, and ZL provided technical assistance. FW and ZL wrote the manuscript. All authors read and approved the final manuscript.

## Conflict of Interest

The authors declare that the research was conducted in the absence of any commercial or financial relationships that could be construed as a potential conflict of interest.
